# Attitudes, barriers, and facilitators toward tools supporting appropriate prescribing among healthcare professionals: a cross-sectional study

**DOI:** 10.1007/s00228-025-03852-4

**Published:** 2025-05-31

**Authors:** Monia Donati, Valentina Giunchi, Giulia Grillini, Marco Domenicali, Maria Lia Lunardelli, Veronica Pasini, Susy Milandri, Monica Mussoni, Fabio Pieraccini, Elisa Sangiorgi, Emanuel Raschi, Valentina Colonnello, Carlotta Lunghi, Elisabetta Poluzzi

**Affiliations:** 1 Department of Medical and Surgical Sciences, University of Bologna, Bologna, Italy; 2 Geriatric Acute Care, Orthogeriatric Unit & Center for Diagnosis of Cognitive Disorders and Dementia, IRCCS-AOUBO, Bologna, Italy; 3 General Practitioner, Cesena District, AUSL Romagna, Cesena, Italy; 4 Local Health Authority of Romagna, Forlì, Italy; 5 Pharmaceutical Care Department, Local Health Authority of Romagna, Forlì, Italy; 6 Drug and Medical Devices Area, Hospital Assistance Sector of the General Directorate for Personal Care, Emilia Romagna Region, Bologna, Italy; 7 Department of Life Sciences, Health and Health Professions, Link Campus University, Rome, Italy

**Keywords:** Appropriate prescribing, Drug-drug interactions, Healthcare professionals, Medication safety, Polypharmacy, Prescribing tools

## Abstract

**Purpose:**

Potentially inappropriate prescriptions are associated with an increased risk of drug-drug interactions, adverse events, and unfavorable clinical outcomes, especially in older adults. Although different tools to improve appropriate prescribing have been developed to support healthcare professionals, their application and the barriers to their use remain insufficiently explored. This study aimed to assess Italian healthcare professionals’ knowledge of these tools and identify obstacles to their adoption.

**Methods:**

The study used a purposefully designed questionnaire to assess knowledge, adoption, and barriers related to appropriateness tools. The tools included were identified through a literature review and subsequently refined via expert consensus. Open-ended responses were analyzed using a conventional content analysis approach, and the analyses focused on differences across professional groups.

**Results:**

The survey collected 657 responses from pharmacists (35%), nurses (26%), general practitioners (22%), geriatricians/internists (9%), and other physicians (8%). The Beers and STOPP/START criteria were used by 38% and 34% of participants, respectively, with geriatricians and other physicians being the primary users. Additionally, 34% of participants reported using specific software integrated into their institutional computer systems. Among 294 respondents identifying barriers to appropriate prescribing, the most common were lack of time (14%), lack of knowledge (10%), and accessibility/costs of digital tools (8%). Key facilitators included specific training (38%), integrated software/apps (29%), and more time with patients (11%).

**Conclusions:**

The adoption of tools supporting appropriate prescribing remains limited among healthcare professionals in Italy, with significant differences among professionals. Policymakers and healthcare institutions should focus on education, interprofessional collaboration, and user-friendly digital solutions to improve prescribing process and patient safety.

**Supplementary Information:**

The online version contains supplementary material available at 10.1007/s00228-025-03852-4.

## Introduction

Optimizing prescription appropriateness is crucial for enhancing patient safety, reducing adverse drug events, and improving healthcare efficiency worldwide. Harvey [23] defined appropriate care as a “strategy of action which maximizes the potential health benefits valued by informed individuals or populations after considering the likely outcomes, their probabilities and their cost.” Similarly, appropriate prescribing aims to balance efficacy, safety, cost, and patient preferences [7]. In contrast, inappropriate prescribing, such as under-prescribing, over-prescribing, prescribing cascades, and the prescription of potentially inappropriate medications (PIMs), represents a healthcare concern [42]. Inappropriate prescribing can have serious consequences for patients, such as an increased risk of drug-drug interactions, adverse events, hospitalizations, and mortality, while also raising healthcare costs [18, 19, 24, 26, 29, 32, 38]. Older adults and multimorbid patients are particularly vulnerable due to frequent polypharmacy, which exacerbates these risks [47]. Moreover, inappropriate prescribing, often compounded by inadequate communication between prescribers (e.g., specialists and family clinicians), is associated with poor medication adherence [41], leading to serious health consequences for the patient, such as reduced quality of life and lower survival rates [34].

Polypharmacy, especially in aging populations, increases the risk of inappropriate prescribing. This issue is particularly relevant in southern Europe, where demographic trends and healthcare practices amplify the challenge [8, 10, 37]. In Italy, studies have highlighted variations in the prevalence of inappropriate prescriptions across healthcare settings [11, 21, 36, 39] and the effectiveness of available appropriateness tools [13, 16, 17]. Various tools have been developed to improve prescription appropriateness [6], including the Beers criteria 5, the Screening Tool of Older Persons’ Potentially Inappropriate Prescriptions (STOPP), and the Screening Tool to Alert Doctors to the Right Treatment (START) [40], and Computerized Decision Support Systems (CDSS), which are digital tools that integrate clinical guidelines with patient data to support prescribing decisions [3, 28, 43]. These tools are referred to as “appropriateness tools” throughout the paper, as they support healthcare professionals in evaluating, optimizing, or discontinuing pharmacological treatments to improve prescribing appropriateness in clinical practice. These tools provide healthcare professionals with a standardized approach to identify medications that may pose risks or have limited benefits in older individuals [33]. However, despite their potential benefits, their adoption in clinical practice remains inconsistent among healthcare professionals. Although some barriers to the use of these tools have been previously identified—such as time constraints, insufficient training, and technological limitations [15, 30, 46]—most studies focused on prescribing physicians or only on digital tools. To our knowledge, no study has systematically explored the perspective of different healthcare professionals—not only physicians—on both the use of traditional and digital tools, including implicit and explicit criteria, to enhance appropriate prescribing. Furthermore, only a few have identified potential barriers and facilitators across different healthcare professionals.

This study aimed to assess healthcare professionals’ awareness, adoption, and perceived barriers to prescription appropriateness tools, as well as to identify potential facilitators to enhance their implementation in routine practice across professional groups.

## Materials and methods

### Questionnaire creation

We developed an anonymous web-based questionnaire for healthcare professionals across Italy, regardless of the care setting. The questionnaire was structured into three sections. The first section covered demographic and occupational information. The second explored participants’ attitudes towards tools designed to improve patients’ medication adherence, and the third focused on tools related to prescribing appropriateness (Supplementary material). Additionally, to ensure the completeness of the information gathered, a question on other possible known tools for supporting appropriate prescribing that were not mentioned in the questionnaire and used in the last 12 months, alongside an open-ended question on limitations encountered in the use of appropriateness tools and on future perspectives on their use, was added. Given the distinct focus of the adherence section, only sections one and three were analyzed in this manuscript. Findings from section two will be reported separately.

The list of tools included in section three on prescribing appropriateness was derived from a PubMed literature review conducted up to January 2023 to identify the most commonly used tools for enhancing prescribing appropriateness (Tables S1 and S2). Subsequently, a panel of seven expert healthcare professionals (one internist, one geriatrician, one general practitioner, one nurse, and three pharmacists) validated this list to ensure its relevance in the Italian healthcare context and evaluated the questionnaire to ensure completeness. They excluded tools specific to niche settings in favor of those more broadly applicable to Italian routine clinical practice.

### Questionnaire distribution

The final version of the questionnaire was distributed online using Microsoft Forms^®^ between October and December 2023. Participants were recruited using a snowball sampling method, starting from a panel of seven healthcare professionals involved in the questionnaire evaluation process. These experts—representing different settings and professions (e.g., internist, geriatrician, pharmacist, nurse, GP)—were asked to disseminate the survey through their existing networks, including hospital mailing lists, institutional and regional contacts, and professional communication groups (e.g., email and WhatsApp). This decentralized approach aimed to ensure participant anonymity while capturing a broad and diverse sample. No social media channels were used.

### Human ethics and consent to participate declarations

Ethical consent for this study was obtained on October 9, 2023, from the University of Bologna Bioethical Committee (Prot. n. 0290273 del 09/10/2023). In line with the Bioethical Committee’s requirements, each respondent was asked for their consent before accessing the questionnaire. If consent was not granted, the questionnaire would not proceed further.

### Statistical analysis

Descriptive statistics were generated, summarizing categorical variables with absolute and percentage frequencies, and continuous variables with the median and interquartile range (Q1–Q3). Depending on the data distribution, the Chi-square test or Fisher’s exact test was employed to compare tool usage across different professions (*p*-values were simulated through 2000 iterations when expected frequencies were too small for accurate *p*-value computation). The Bonferroni correction was applied to adjust for multiple comparisons.

The open-ended responses were systematically analyzed using a multi-step conventional content analysis approach [25]. First, the responses were read repeatedly to gain an overall understanding of the content. Each response was then examined word by word to identify codes, with keywords or phrases representing important concepts highlighted. After deriving the codes directly from the text, the relationships between them were explored to develop broader categories. To ensure reliability, each response was categorized and coded independently by two researchers (CL and MD). Any discrepancies in coding were resolved through a reconciliation process involving a third reviewer (VC). This collaborative approach ensured that all key concepts emerging from the data were captured comprehensively, minimizing bias and enhancing the rigor of the analysis. Any ambiguous or unclear responses were discussed within the research team to determine the most appropriate categorization. Additionally, the occurrence of each category was quantified to assess the absolute and relative frequency of the identified concepts. The final interrater reliability between the two raters was excellent (with the interrater agreement coefficient Cohen κ > 0.80).

## Results

Out of the 680 responses collected, 20 respondents did not consent to data treatment, resulting in 660 valid responses for the analysis. Most of them were women (67%), aged between 40 and 60 (54% vs. 29% under 40, and 17% over 60), and working as pharmacists (35%), nurses (26%), and general practitioners (GPs, 22%). The median years of professional experience varied significantly by profession (*p* value < 0.001): other MDs reported the highest professional experience (median: 20 years; IQR, 11.8-32.3), while GPs had the lowest experience (10; 2–28). The number of patients seen weekly also differed by profession (*p* value < 0.001). Pharmacists reported the highest number of patients (median, 300; IQR, 150–500), 175 of whom were aged over 65 years old. Across all categories of involved health professionals, more than half of the patients receiving care were aged 65 and older (Table 1).

**Table 1:** Demographic and occupational characteristics of the respondents (*N* = 660)

Characteristics	All	General practitioner	Geriatrician/internist	Other MD	Pharmacist	Nurse	Other	*p*-value	*q*-value
	660	142 (21.52%)	57 (8.64%)	56 (8.48%)	229 (34.70%)	173 (26.21%)	3 (0.45%)		
**Age**								< 0.001	< 0.001
< 40 years	188 (28.48%)	41 (28.87%)	18 (31.58%)	7 (12.50%)	62 (27.07%)	60 (34.68%)			
40–60 years	359 (54.39%)	53 (37.33%)	28 (49.12%)	31 (55.36%)	137 (59.83%)	107 (61.85%)			
> 60 years	113 (17.12%)	48 (33.80%)	11 (19.30%)	18 (32.14%)	30 (13.10%)	6 (3.47%)			
**Gender**								< 0.001	< 0.001
Female	442 (67.07%)	73 (51.77%)	32 (56.14%)	29 (51.79%)	156 (68.12%)	150 (86.71%)			
Male	217 (32.93%)	68 (48.23%)	25 (43.86%)	27 (48.21%)	73 (31.88%)	23 (13.29%)			
Not specified	1	1							
**Work experience** (years) median (Q1–Q3)								< 0.001	< 0.001
	16 (6–27)	10 (2–28)	13 (7–23)	20 (11.75–32.25)	18 (9–27)	17 (6–27.25)			
**No. of patients per week **median (Q1–Q3)								< 0.001	< 0.001
	80 (40–230)	100 (60–130)	30 (25–50)	30 (20–50)	300 (150–500)	45 (30–70)			
**No. of older patients per week **median (Q1–Q3)								< 0.001	< 0.001
	60 (30–140)	60 (40–85)	30 (20–45)	20 (10–35)	175 (80–300)	40 (22.25–63.25)			

*MD* medical doctor, *Q1* first quartile, *Q3* third quartile. The *q*-value represents the *p*-value adjusted using the Bonferroni correction for multiple testing

### Usage and awareness of appropriateness tools

**Fig. 1:**
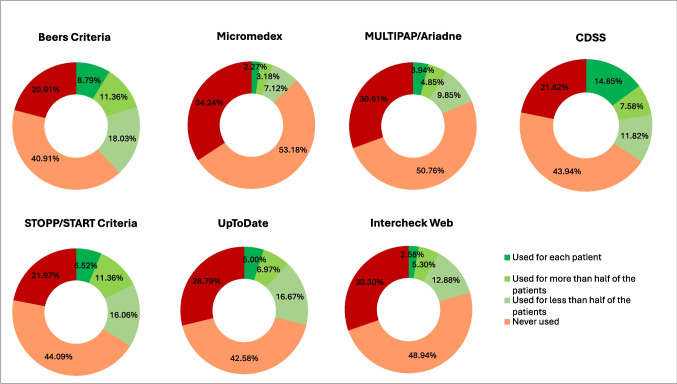
Usage and knowledge of each appropriateness tool

The majority of respondents reported either never using (40.9 to 53.2%) or being unaware (20.9 to 34.2%) of appropriateness tools (Fig. 1). The Beers and STOPP/START criteria were among the most frequently used tools. Specifically, 8.8% of respondents reported using the Beers criteria for every patient, while 11.4% used them for more than half of their patients. Similarly, 6.5% of respondents indicated they used the STOPP/START criteria for every patient, with 11.4% using them for more than half of their patients. In contrast, the MULTIPAP/Ariadne principles had the lowest usage, with the majority of respondents either never using them (53.2%) or being unaware of them (30.6%). Similar usage patterns were observed to other tools like Micromedex (http://www.micromedexsolutions.com/) and UpToDate (https://www.uptodate.com/). Also, the Italian INTERCheck Web tool [22] was never used by 48.9% of respondents, and 30.3% were unaware of it. CDSS were relatively more widely adopted compared to other tools. About 14.9% of respondents used CDSS for every patient, and 7.6% used them for more than half of their patients (Fig. 1).

### Variations by profession

**Fig. 2:**
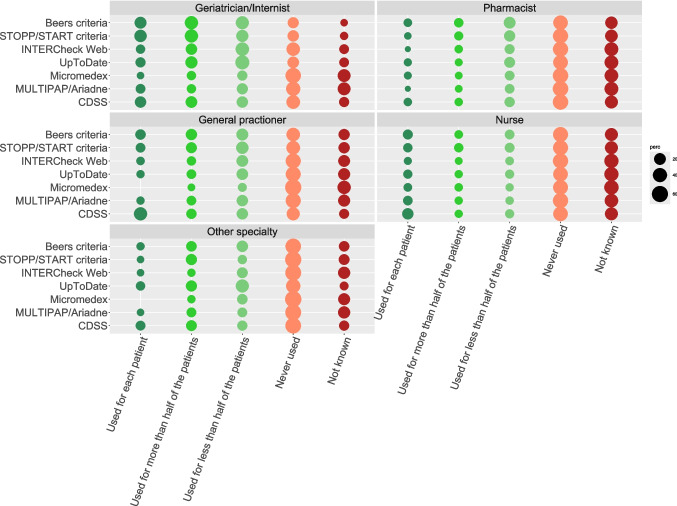
Use of each appropriateness tool by profession

Before conducting the profession-specific analysis, three respondents categorized as “other profession” were excluded due to their heterogeneity and small number, making them unsuitable for this analysis. Geriatricians and internists emerged as the most frequent users of appropriateness tools. Specifically, 56.1% of them reported using the STOPP/START criteria for at least half of their patients, while 52.6% reported using the Beers criteria and 33.3% UpToDate. Additionally, 36.8% of geriatricians and internists reported using CDSS for at least half of their patients. CDSS were also frequently used by GPs, with 42.3% reporting use for at least half of their patients. In contrast, other professions, such as pharmacists or nurses, reported much lower usage rates of these appropriateness tools. For example, only 3.9% of pharmacists reported using CDSS for each patient, and 43.9% of nurses never used these systems (Fig. 2 and Table S3).

The use of appropriateness tools was inversely related to years of professional experience. Healthcare professionals with fewer years of experience were more likely to use tools such as the STOPP/START criteria, UpToDate, INTERCheck Web, and CDSS systems. For example, those using CDSS for every patient had a median of 11 years (3, 22) of experience, while those never using them had a median of 18 years (9, 29) (Table S4).

### Barriers to tool adoption and facilitators of appropriate prescribing

A total of 291 participants (44%) answered the open-ended question on the limitations hindering the use of appropriateness tools. More than half of them reported no obstacles, with differences among health professionals ranging from 52% among geriatricians and internists to 72% among nurses. Among those who reported barriers, the most commonly reported limitation was a lack of time (13.9%), followed by a lack of knowledge or familiarity with the tools (9.6%), poor dissemination and usability of information systems (8.2%), and costs and technical difficulties associated with digital tools (6.4%). Barriers varied among professions: lack of time was the more frequently reported by geriatricians and internists (20.7%), GPs (18.3%), and pharmacists (12.8%), while other MDs and nurses highlighted lack of knowledge or familiarity with the tools (16.2% and 9.3%, respectively) (Table 2).

**Table 2:** Reported barriers to the use of appropriateness tools and proposed solutions to improve appropriate prescribing, according to the profession

	All	Geriatrician/internist	General practitioner	Other MD	Pharmacist	Nurse	*p***-**value	*q***-**value
**Barriers**							0.6	1
None	165 (58.93%)	15 (51.72%)	43 (52.44%)	21 (56.76%)	47 (60.26%)	39 (72.22%)		
Lack of time	39 (13.93%)	6 (20.69%)	15 (18.29%)	5 (13.51%)	10 (12.82%)	3 (5.56%)		
Lack of knowledge or familiarity with the tool	27 (9.64%)	1 (3.45%)	6 (7.32%)	6 (16.22%)	9 (11.54%)	5 (9.26%)		
Dissemination and usability of information systems	23 (8.21%)	4 (13.79%)	9 (10.98%)	3 (8.11%)	3 (3.85%)	4 (7.41%)		
Cost and ease of use of digital tools	18 (6.43%)	3 (10.34%)	6 (7.32%)	1 (2.70%)	6 (7.69%)	2 (3.70%)		
Poor motivation	3 (1.07%)	0 (0.00%)	2 (2.44%)	0 (0.00%)	1 (1.28%)	0 (0.00%)		
Other	5 (1.79%)	0 (0.00%)	1 (1.22%)	1 (2.70%)	2 (2.56%)	1 (1.85%)		
**Proposed solutions**							0.020	0.143428
Implementation of digital tools	110 (37.54%)	9 (36.00%)	38 (44.71%)	14 (43.75%)	32 (34.41%)	17 (29.31%)		
Education	86 (29.35%)	9 (36.00%)	17 (20.00%)	11 (34.38%)	27 (29.03%)	22 (37.93%)		
Time	31 (10.58%)	1 (4.00%)	15 (17.65%)	5 (15.63%)	8 (8.60%)	2 (3.45%)		
Interprofessional collaboration	22 (7.51%)	4 (16.00%)	4 (4.71%)	0 (0.00%)	9 (9.68%)	5 (8.62%)		
Guidelines	12 (4.10%)	1 (4.00%)	5 (5.88%)	0 (0.00%)	4 (4.30%)	2 (3.45%)		
Medication review	11 (3.75%)	0 (0.00%)	1 (1.18%)	0 (0.00%)	4 (4.30%)	6 (10.34%)		
Patient/caregiver collaboration	10 (3.41%)	0 (0.00%)	2 (2.35%)	2 (6.25%)	6 (6.45%)	0 (0.00%)		
Legal protection for professionals	2 (0.68%)	1 (4.00%)	1 (1.18%)	0 (0.00%)	0 (0.00%)	0 (0.00%)		
Other	9 (3.07%)	0 (0.00%)	2 (2.35%)	0 (0.00%)	3 (3.23%)	4 (6.90%)		

*Q*-value stands for the *p*-value corrected for multiple testing through the Bonferroni correction. The column “All” is not included in the statistical comparison

A total of 369 participants (56%) answered the open-ended question to propose facilitators to improve appropriateness. Seventeen responses were excluded as irrelevant. The most reported proposed solutions were the implementation of digital tools (37.5%), enhanced education and training (29.4%), allocating more time for each patient (10.6%), and interprofessional collaboration (7.51%), with no significant differences observed between healthcare professions (Table 2).

### Other appropriateness tools

The open-ended question on the use of appropriateness tools beyond those specified in the questionnaire was answered by 411 participants (62%); 121 responses were excluded as invalid. Workplace information systems (23%) and Websites/Apps (12%) emerged as the most used additional tools (Table S5).

## Discussion

This study provides insights into the awareness, adoption, and challenges associated with prescribing appropriateness tools among healthcare professionals from diverse backgrounds. Findings highlight variations in tool utilization across different professional groups, the barriers hindering their implementation (including time constraints, training gaps, and digital accessibility) and the need for strategies to enhance their adoption. These insights contribute to the broader global discussion on optimizing prescribing practices and integrating decision-support tools into routine clinical workflows.

### Knowledge and usage of appropriateness tools

A key finding of this survey is that appropriateness tools remain underutilized across all healthcare professions, with geriatricians and internists reporting the highest use, particularly of Beers and STOPP/START criteria. GPs showed greater confidence in digital tools, while pharmacists and nurses reported lower usage rates overall, except for CDSS. Although international data on the prevalence of use of appropriateness tools among healthcare professionals are limited, our findings align with existing studies that reported low awareness, limited knowledge, and scarce utilization of such tools [1, 2, 4, 44]. This emphasizes the importance of educational initiatives and implementation programs within clinical practice to promote appropriate drug prescriptions. Furthermore, variability in professional engagement in medication prescribing suggests a need for greater interdisciplinary collaboration. Internationally, prescribing responsibilities vary across healthcare systems. In many countries, prescribing authority is restricted to physicians, while pharmacists and nurses do not have the jurisdiction to prescribe medications but play a key role in medication management and patient safety. Their regular interactions with patients position them as essential actors in monitoring medication adherence, identifying adverse drug reactions, and supporting deprescribing efforts. For instance, in home-based care settings, nurses are uniquely placed to assess self-medication practices and ensure patients’ adherence to prescribed therapeutic regimens. Similarly, pharmacists, with their expertise in drug interactions and patient education, can support safer prescribing through medication reviews and deprescribing [14]. Furthermore, pharmacists can assist patients in improving adherence and support physicians in deprescribing efforts. Evidence from international settings suggests that pharmacist-led medication review programs can reduce inappropriate prescribing and hospital readmission [12, 45].

The involvement of nurses and pharmacists is particularly important in addressing self-prescription, including unsupervised use of over-the-counter (OTC) drugs (e.g., NSAIDs and proton pump inhibitors), which may increase the risk of interactions and adverse events if prescribers are unaware of their use. This concern is not unique to Italy—many health systems struggle with inadequate documentation of OTC medication use, leaving prescribers unaware of potentially harmful interactions. The 2024 Italian Report on Medicines Use (*Osservatorio Nazionale sull’Impiego dei Medicinali*—OsMed) from the Italian Medicines Agency (*Agenzia Italiana del Farmaco*—AIFA) highlighted this issue, emphasizing the urgent need for strategies to integrate self-medication monitoring into clinical workflows (Italian Medicines Agency, 2024, 2023). Digital solutions, such as mobile apps that track medication regimens, have gained traction internationally as a means to bridge communication gaps between patients and healthcare providers. In Italy, the AIFA mobile app (https://www.aifa.gov.it/mobile-app-aifa) serves this function by improving patient education and awareness regarding medication and supporting self-monitoring.

### Barriers to appropriateness tool adoption and proposed solutions

Key barriers to the adoption of appropriateness tools included time constraints, insufficient knowledge or familiarity, and challenges in the usability and dissemination of digital systems. These barriers were more frequently reported by non-prescribing professionals, such as pharmacists and nurses, highlighting the need for targeted education, better integration of digital tools, and enhanced interprofessional collaboration.

**Fig. 3:**
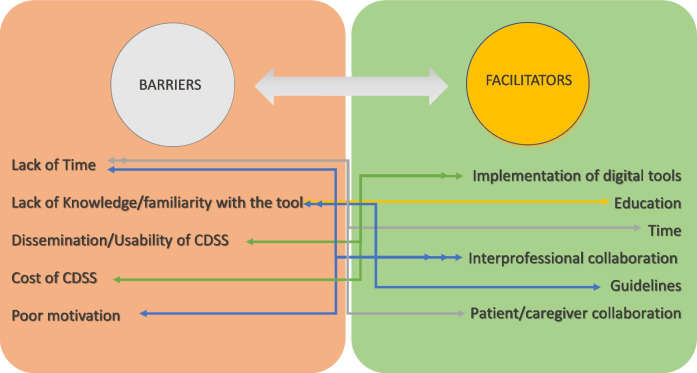
Interplay between barriers and proposed solutions (facilitators)

Participants emphasized the need for better education and broader adoption of digital tools (Fig. 3). Effective implementation requires equipping professionals with the necessary knowledge and skills. Training programs aimed at improving awareness and competency in using these tools could significantly enhance their adoption and impact in clinical practice. The potential of digital medicine to enhance prescribing practices warrants significant consideration. As healthcare systems increasingly adopt digital health solutions, tools that integrate real-time patient data with clinical guidelines have the capacity to support decision-making processes and ensure more appropriate prescribing.

A lack of time was another widely reported barrier. Increasing the time available for each patient and for training is crucial to support appropriate prescribing. This issue likely reflects the strain on an overburdened national health service, marked by regional and territorial disparities alongside daily challenges such as prolonged waiting times, which inevitably disrupt the effective management of patients’ care pathways. At the forefront, primary care is particularly overwhelmed, facing an aging patient population, insufficient infrastructures, and limited access to specialized professionals (Fondazione GIMBE, 2024). In this regard, incorporating digital tools into clinical practice could bridge the time gap by accelerating prescribing process assessments.

Another critical aspect of appropriate prescribing is transitioning from one care service to another, particularly for older patients. Effective communication between hospital specialists (e.g., geriatricians or internists) and GPs is essential during this stage to ensure proper continuity of care. A recent survey conducted in Denmark [31] revealed that general practitioners often feel left alone to manage polypharmacy initiated in hospital settings, underscoring the importance of structured collaboration across different healthcare environments.

Multidisciplinary teams of healthcare professionals—including physicians, nurses, and pharmacists—represent a crucial objective in the healthcare setting and could act as both a driver for the active involvement of all professionals responsible for patient care and a viable strategy to alleviate the burden of individual healthcare providers. Enhanced communication among healthcare professionals fosters a more comprehensive and personalized approach to medication management, ultimately improving patient outcomes. Such teamwork is essential, particularly in managing polypharmacy, a common challenge in older adults [9, 35]. Finally, promoting dialogue among professionals may help address the poor motivation to engage in appropriate prescribing, as reported by some participants.

### Strengths and limitations

To our knowledge, this is the first study to examine healthcare professionals’ perceptions of the tools used to enhance prescription appropriateness in Italy, offering insights that may influence further research and inform policy decisions within healthcare practices. Recruiting a diverse cohort of healthcare professionals facilitated the collection of a broad spectrum of experiences and opinions. Furthermore, integrating both quantitative and qualitative data through structured survey responses and open-ended questions afforded a comprehensive, multidimensional perspective on the knowledge and utilization of prescription appropriateness tools.

Despite these strengths, the study is not without limitations. The reliance on self-reported data may introduce biases; respondents may not accurately reflect their actual practices due to recall or social desirability biases, potentially skewing data related to tool usage and perceived barriers. However, the generally low level of knowledge and use of the appropriateness tools reported by respondents may indicate that such biases have only minimally affected the study results. Additionally, while the findings offer significant insights into the entire Italian context, they might not directly apply to the whole Italian context or other settings with different healthcare infrastructures, professional roles, or cultural attitudes toward prescription management. Nevertheless, we believe that the themes and challenges identified, such as time constraints, limited training, and low level of tool adoption, are common across healthcare systems and may hold relevance beyond the Italian context. Furthermore, the snowball sampling technique did not allow for determining the response rate, thereby hindering a comprehensive understanding of potential non-response bias, which could impact the representativeness and accuracy of the findings. Finally, even if the questionnaire was not formally validated, it was thoroughly refined based on feedback from a panel of healthcare professionals and adapted to better align with the practical realities and nuances specific to the Italian healthcare context, ensuring its relevance and applicability.

## Conclusions

To summarize, the main findings of this study are outlined below:

- Awareness and use of appropriateness tools varied across healthcare professionals, with geriatricians and internists reporting the highest use, and pharmacists and nurses the lowest.
- Digital tools were more commonly used by general practitioners, while traditional tools like STOPP/START and Beers criteria were more frequently used by geriatricians.
- The main barriers reported were time constraints, limited training, and restricted access to digital systems.
- Facilitators included specific education, integration of tools into clinical software, and increased consultation time.

To address these challenges, policymakers and healthcare institutions should prioritize educational initiatives, interprofessional collaboration, and user-friendly, integrated digital solutions. Further research is needed to explore strategies for implementing and evaluating the impact of appropriateness tools on patient outcomes and healthcare costs. Increasing familiarity with and use of these tools is essential for enhancing patient safety and care quality, both in Italy and globally.

## Supplementary Information

Below is the link to the electronic supplementary material.

ESM 1 (DOCX 334 KB)

## Data Availability

Questionnaire data are anonymized at the source and are available upon written request to the corresponding author.
