# Supplementary material for: Attitudes, barriers, and facilitators toward tools supporting appropriate prescribing among healthcare professionals: a cross-sectional study
Source: Eur J Clin Pharmacol. 2025 May 31;81(8):1155–65. doi: 10.1007/s00228-025-03852-4 (PMC12279595; doi:10.1007/s00228-025-03852-4)
Supplement: Supplementary file 1 — (DOCX 334 KB) [file 228_2025_3852_MOESM1_ESM.docx]

**Supplementary material**

**Attitudes, Barriers, and Facilitators Toward Prescribing Tools Among Healthcare Professionals: A Cross-Sectional Study**

Monia Donati, Valentina Giunchi, Giulia Grillini, Marco Domenicali, Maria Lia Lunardelli, Veronica Pasini, Susy Milandri, Monica Mussoni, Fabio Pieraccini, Elisa Sangiorgi, Emanuel Raschi, Valentina Colonnello, Carlotta Lunghi, Elisabetta Poluzzi

**Table S1**– Terms included in the PubMed research query for appropriateness tools

| MeSH | “Drug Prescriptions”,  “Inappropriate Prescribing”,  “Decision Support Systems, Clinical” |
| --- | --- |
| Keyword | “inappropriate”, “inappropriateness”, “appropriateness”  AND  “medication”, “drug”, “prescribing”, “presciption*”  AND  “intervention*” |

**Table S2** – Articles retrieved in the literature review along with the reported appropriateness tools

| Tool | Article | Authors | Year |
| --- | --- | --- | --- |
| E-health records, Computerized alert system | McKibben S, De Simoni A, Bush A, Thomas M, Griffiths C. The use of electronic alerts in primary care computer systems to identify the excessive prescription of short-acting beta2-agonists for people with asthma: a systematic review. NPJ Prim Care Respir Med. 2018;28(1):14. Published 2018 Apr 16. doi:10.1038/s41533-018-0080-z | Shauna McKibben | 2018 |
| CPOE, CDSS, E-reminds, Computerized alert system | Hajesmaeel Gohari S, Bahaadinbeigy K, Tajoddini S, R Niakan Kalhori S. Effect of Computerized Physician Order Entry and Clinical Decision Support System on Adverse Drug Events Prevention in the Emergency Department: A Systematic Review. J Pharm Technol. 2021;37(1):53-61. doi:10.1177/8755122520958160 | Sadrieh Hajesmaeel Gohari | 2020 |
| CDSS, Beers Criteria, STOPP/START Criteria | Monteiro L, Maricoto T, Solha IS, Monteiro-Soares M, Martins C. Computerised decision to reduce inappropriate medication in the elderly: a systematic review with meta-analysis protocol. BMJ Open. 2018 Jan 30;8(1):e018988. doi: 10.1136/bmjopen-2017-018988. PMID: 29382677; PMCID: PMC5829874. | Tiago Maricoto | 2018 |
| INTERCheck software, Drug-PIN software | Martocchia A, Bruscia C, Conforti G, et al. Comparison of Computerized Prescription Support Systems in COVID-19 Patients: INTERCheck and Drug-PIN. SN Compr Clin Med. 2022;4(1):3. doi:10.1007/s42399-021-01079-9 | Antonio Martocchia | 2022 |
| CPOE, CDSS, E-reminds, Computerized alert system | Schedlbauer A, Prasad V, Mulvaney C, et al. What evidence supports the use of computerized alerts and prompts to improve clinicians' prescribing behavior?. J Am Med Inform Assoc. 2009;16(4):531-538. doi:10.1197/jamia.M2910 | Angela Schedlbauer | 2009 |
| INTERCheck software | Martocchia A, Spuntarelli V, Aiello F, et al. Using INTERCheck® to Evaluate the Incidence of Adverse Events and Drug-Drug Interactions in Out- and Inpatients Exposed to Polypharmacy. Drugs Real World Outcomes. 2020;7(3):243-249. doi:10.1007/s40801-020-00193-9 | Antonio Martocchia | 2020 |
| CPOE, CDSS | Manias E, Williams A, Liew D. Interventions to reduce medication errors in adult intensive care: a systematic review. Br J Clin Pharmacol. 2012 Sep;74(3):411-23. doi: 10.1111/j.1365-2125.2012.04220.x. PMID: 22348303; PMCID: PMC3477343. | Elizabeth Manias | 2012 |
| CDSS | Marasinghe KM. Computerised clinical decision support systems to improve medication safety in long-term care homes: a systematic review. BMJ Open. 2015;5(5):e006539. Published 2015 May 12. doi:10.1136/bmjopen-2014-006539 | Keshini Madara Marasinghe | 2015 |
| CPOE, CDSS | van der Linden CM, Jansen PA, Grouls RJ, et al. Systems that prevent unwanted represcription of drugs withdrawn because of adverse drug events: a systematic review. Ther Adv Drug Saf. 2013;4(2):73-90. doi:10.1177/2042098613477125 | Carolien M.J | 2013 |
| CPOE, CDSS | Kawamoto K, Houlihan CA, Balas EA, Lobach DF. Improving clinical practice using clinical decision support systems: a systematic review of trials to identify features critical to success. BMJ. 2005;330(7494):765. doi:10.1136/bmj.38398.500764.8F | Kensaku Kawamoto | 2005 |
| CDSS | Pearson SA, Moxey A, Robertson J, et al. Do computerised clinical decision support systems for prescribing change practice? A systematic review of the literature (1990-2007). BMC Health Serv Res. 2009;9:154. Published 2009 Aug 28. doi:10.1186/1472-6963-9-154 | Sallie-Anne Pearson | 2009 |
| CDSS | Hemens BJ, Holbrook A, Tonkin M, et al. Computerized clinical decision support systems for drug prescribing and management: a decision-maker-researcher partnership systematic review. Implement Sci. 2011;6:89. Published 2011 Aug 3. doi:10.1186/1748-5908-6-89 | Brian J Hemens | 2011 |
| STOPP/START criteria, STRIP (Systematic Tool to Reduce Inappropriate Prescribing), TRIM (Tool to Reduce Inappropriate Medications), MERIS algorithm, NORGEP-NH (Norwegian General Practice – Nursing Home criteria), Computerized alert system | Atmaja DS, Yulistiani, Suharjono, Zairina E. Detection tools for prediction and identification of adverse drug reactions in older patients: a systematic review and meta-analysis. Sci Rep. 2022;12(1):13189. Published 2022 Aug 1. doi:10.1038/s41598-022-17410-w | Dewi SusantiAtmaja | 2022 |
| CDSS | Damoiseaux-Volman BA, Medlock S, van der Meulen DM, et al. Clinical validation of clinical decision support systems for medication review: A scoping review. Br J Clin Pharmacol. 2022;88(5):2035-2051. doi:10.1111/bcp.15160 | Birgit A. Damoiseaux-Volman | 2021 |
| AMS Program (antimicrobial stewardship programme) | Helou RI, Foudraine DE, Catho G, Peyravi Latif A, Verkaik NJ, Verbon A. Use of stewardship smartphone applications by physicians and prescribing of antimicrobials in hospitals: A systematic review. PLoS One. 2020;15(9):e0239751. Published 2020 Sep 29. doi:10.1371/journal.pone.0239751 | R. I. Helou | 2020 |
| Educational material, awareness campaigns, audit feedback | Nguyen T, Nguyen HQ, Widyakusuma NN, Nguyen TH, Pham TT, Taxis K. Enhancing prescribing of guideline-recommended medications for ischaemic heart diseases: a systematic review and meta-analysis of interventions targeted at healthcare professionals. BMJ Open. 2018 Jan 10;8(1):e018271. doi: 10.1136/bmjopen-2017-018271. PMID: 29326185; PMCID: PMC5988110. | Thang Nguyen | 2018 |
| STOPP/START criteria, Beers criteria, NORGEP-NH (Norwegian General Practice – Nursing Home criteria), Laroche List, Holmes criteria | Hukins D, Macleod U, Boland JW. Identifying potentially inappropriate prescribing in older people with dementia: a systematic review. Eur J Clin Pharmacol. 2019;75(4):467-481. doi:10.1007/s00228-018-02612-x | Deborah Hukins | 2019 |
| Beers criteria, Holmes criteria, MAI (Medication Appropriateness Index) | Cadogan CA, Murphy M, Boland M, Bennett K, McLean S, Hughes C. Prescribing practices, patterns, and potential harms in patients receiving palliative care: A systematic scoping review. Explor Res Clin Soc Pharm. 2021 Jul 23;3:100050. doi: 10.1016/j.rcsop.2021.100050. PMID: 35480601; PMCID: PMC9031741. | Cathal A. Cadogan | 2021 |
| Beers criteria, FORTA list | Murphy M, Bennett K, Ryan S, Hughes CM, Lavan AH, Cadogan CA. A systematic scoping review of interventions to optimise medication prescribing and adherence in older adults with cancer. Res Social Adm Pharm. 2022;18(3):2392-2402. doi:10.1016/j.sapharm.2021.04.011 | Melanie Murphy | 2022 |
| Beers criteria, STOPP/START criteria, Micromedex, Medscape, Drugs.com, Lexicomp ONCOFARMA | Ayalew MB, Spark MJ, Quirk F, Dieberg G. Potentially inappropriate prescribing for adults living with diabetes mellitus: a scoping review. Int J Clin Pharm. 2022;44(4):860-872. doi:10.1007/s11096-022-01414-7 | Mohammed Biset Ayalew | 2022 |
| PINCER (Information Technology Intervention for Medication Errors), iMPACT (Investigation Prescription Picking Accuracy for Critical Error Types), SMASH (Salford Medication Safety Dashboard) | Khawagi WY, Steinke DT, Nguyen J, Keers RN. Identifying potential prescribing safety indicators related to mental health disorders and medications: A systematic review. PLoS One. 2019;14(5):e0217406. Published 2019 May 24. doi:10.1371/journal.pone.0217406 | Wael Y. Khawagi | 2019 |
| INTERCheck software | Petrini E, Caviglia GP, Pellicano R, Saracco GM, Morino M, Ribaldone DG. Risk of drug interactions and prescription appropriateness in elderly patients. Ir J Med Sci. 2020;189(3):953-959. doi:10.1007/s11845-019-02148-8 | Elisa Petrini | 2023 |
| CPOE, CDSS | Ciapponi A, Fernandez Nievas SE, Seijo M, et al. Reducing medication errors for adults in hospital settings. Cochrane Database Syst Rev. 2021;11(11):CD009985. Published 2021 Nov 25. doi:10.1002/14651858.CD009985.pub2 | Agustín Ciapponi | 2021 |
| Beers criteria, STOPP/START criteria | Young RA, Fulda KG, Espinoza A, et al. Ambulatory Medication Safety in Primary Care: A Systematic Review. J Am Board Fam Med. 2022;35(3):610-628. doi:10.3122/jabfm.2022.03.210334 | Richard A Young | 2022 |
| Beers criteria, STOPP/START criteria, audit feedback | Lee JQ, Ying K, Lun P, et al. Intervention elements to reduce inappropriate prescribing for older adults with multimorbidity receiving outpatient care: a scoping review. BMJ Open. 2020;10(8):e039543. Published 2020 Aug 20. doi:10.1136/bmjopen-2020-039543 | Jia Qi Lee | 2020 |
| Beers criteria, STOPP/START criteria, PRISCUS list | Mucherino S, Casula M, Galimberti F, et al. The Effectiveness of Interventions to Evaluate and Reduce Healthcare Costs of Potentially Inappropriate Prescriptions among the Older Adults: A Systematic Review. Int J Environ Res Public Health. 2022;19(11):6724. Published 2022 May 31. doi:10.3390/ijerph19116724 | Sara Mucherino | 2022 |
| Beers criteria, STOPP/START criteria, MAI (Medication Appropriateness Index), STRIP (Systematic Tool to Reduce Inappropriate Prescribing), SHiM (Structured HIstory taking of Medication use), CPOE | Lavan AH, Gallagher PF, O'Mahony D. Methods to reduce prescribing errors in elderly patients with multimorbidity. Clin Interv Aging. 2016;11:857-866. Published 2016 Jun 23. doi:10.2147/CIA.S80280 | Amanda H Lavan | 2016 |
| INTERCheck software | Catrini E, Ferrario L, Mazzone A, Varalli L, Gatti F, Cannavacciuolo L, Ponsiglione C, Foglia E. Tools supporting polypharmacy management in Italy: Factors determining digital technologies' intention to use in clinical practice. Health Sci Rep. 2022 May 19;5(3):e647. doi: 10.1002/hsr2.647. PMID: 35601037; PMCID: PMC9117970. | Elisabetta Catrini | 2022 |
| STOPP/START criteria, PRISCUS list, CPOE, CDSS | Johansson T, Abuzahra ME, Keller S, et al. Impact of strategies to reduce polypharmacy on clinically relevant endpoints: a systematic review and meta-analysis. Br J Clin Pharmacol. 2016;82(2):532-548. doi:10.1111/bcp.12959 | Tim Johansson | 2016 |
| STOPP/START criteria, MAI (Medication Appropriateness Index), PAL (Prescription Advantage List), McLeod criteria, CDSS | Cooper JA, Cadogan CA, Patterson SM, et al. Interventions to improve the appropriate use of polypharmacy in older people: a Cochrane systematic review. BMJ Open. 2015;5(12):e009235. Published 2015 Dec 9. doi:10.1136/bmjopen-2015-009235 | Janine A Cooper | 2015 |
| Beers criteria, STOPP/START criteria, PRISCUS list, TRIM (Tool to Reduce Inappropriate Medications), MAI (Medication Appropriateness Index), ACOVE (Assessing Care Of Vulnerable Elderly) quality indicators, FORTA list, CDSS | Rankin A, Cadogan CA, Patterson SM, et al. Interventions to improve the appropriate use of polypharmacy for older people. Cochrane Database Syst Rev. 2018;9(9):CD008165. Published 2018 Sep 3. doi:10.1002/14651858.CD008165.pub4 | Rankin A | 2018 |
| STOPP/START criteria, MAI (Medication Appropriateness Index), DBI (Drug Burden Index) | Croke A, Cardwell K, Clyne B, Moriarty F, McCullagh L, Smith SM. The effectiveness and cost of integrating pharmacists within general practice to optimize prescribing and health outcomes in primary care patients with polypharmacy: a systematic review. BMC Prim Care. 2023;24(1):41. Published 2023 Feb 6. doi:10.1186/s12875-022-01952-z | Aisling Croke | 2023 |
| STOPP/START criteria, MAI (Medication Appropriateness Index), CDSS | Alldred DP, Kennedy MC, Hughes C, Chen TF, Miller P. Interventions to optimise prescribing for older people in care homes. Cochrane Database Syst Rev. 2016;2(2):CD009095. Published 2016 Feb 12. doi:10.1002/14651858.CD009095.pub3 | Alldred DP | 2016 |
| Drug-PIN software | Salamone S, Spirito S, Simmaco M, et al. Prescription Advice Based on Data of Drug-Drug-Gene Interaction of Patients with Polypharmacy. Pharmgenomics Pers Med. 2022;15:765-773. Published 2022 Aug 18. doi:10.2147/PGPM.S368606 | Sandro Salamone | 2022 |
| STOPP/START criteria, ACOVE (Assessing Care Of Vulnerable Elderly) quality indicators | Meid AD, Lampert A, Burnett A, Seidling HM, Haefeli WE. The impact of pharmaceutical care interventions for medication underuse in older people: a systematic review and meta-analysis. Br J Clin Pharmacol. 2015 Oct;80(4):768-76. doi: 10.1111/bcp.12657. Epub 2015 Jun 12. PMID: 25868941; PMCID: PMC4594715. | Andreas D. Meid | 2015 |
| CDSS | Salmasian H, Tran TH, Chase HS, Friedman C. Medication-indication knowledge bases: a systematic review and critical appraisal. J Am Med Inform Assoc. 2015;22(6):1261-1270. doi:10.1093/jamia/ocv129 | Hojjat Salmasian | 2015 |
| STOPP/START criteria, MAI (Medication Appropriateness Index), CDSS | Croke A, Cardwell K, Clyne B, Moriarty F, McCullagh L, Smith SM. The effectiveness and cost of integrating pharmacists within general practice to optimize prescribing and health outcomes in primary care patients with polypharmacy: a systematic review. BMC Prim Care. 2023;24(1):41. Published 2023 Feb 6. doi:10.1186/s12875-022-01952-z | Aisling Croke | 2023 |
| STOPP/START criteria, CDSS | Khalil H, Bell B, Chambers H, Sheikh A, Avery AJ. Professional, structural and organisational interventions in primary care for reducing medication errors. Cochrane Database Syst Rev. 2017;10(10):CD003942. Published 2017 Oct 4. doi:10.1002/14651858.CD003942.pub3 | Khalil H | 2017 |
| CPOE, CDSS | Cuvelier E, Robert L, Musy E, et al. The clinical pharmacist's role in enhancing the relevance of a clinical decision support system. Int J Med Inform. 2021;155:104568. doi:10.1016/j.ijmedinf.2021.104568 | E. Cuvelier | 2021 |
| CDSS, audit feedback | McDonagh MS, Peterson K, Winthrop K, Cantor A, Lazur BH, Buckley DI. Interventions to reduce inappropriate prescribing of antibiotics for acute respiratory tract infections: summary and update of a systematic review. J Int Med Res. 2018;46(8):3337-3357. doi:10.1177/0300060518782519 | Marian S. McDonagh | 2018 |
| CDSS, audit feedback | Xie CX, Chen Q, Hincapié CA, Hofstetter L, Maher CG, Machado GC. Effectiveness of clinical dashboards as audit and feedback or clinical decision support tools on medication use and test ordering: a systematic review of randomized controlled trials. J Am Med Inform Assoc. 2022;29(10):1773-1785. doi:10.1093/jamia/ocac094 | Charis Xuan Xie | 2022 |
| CDSS | Phansalkar S, Desai A, Choksi A, et al. Criteria for assessing high-priority drug-drug interactions for clinical decision support in electronic health records. BMC Med Inform Decis Mak. 2013;13(1):65. Published 2013 Jun 13. doi:10.1186/1472-6947-13-65 | Shobha Phansalkar | 2013 |
| CDSS | Watkins K, Wood H, Schneider CR, Clifford R. Effectiveness of implementation strategies for clinical guidelines to community pharmacy: a systematic review. Implement Sci. 2015;10:151. Published 2015 Oct 29. doi:10.1186/s13012-015-0337-7 | Kim Watkins | 2015 |
| CDSS | Gooch P, Roudsari A. Computerization of workflows, guidelines, and care pathways: a review of implementation challenges for process-oriented health information systems. J Am Med Inform Assoc. 2011 Nov-Dec;18(6):738-48. doi: 10.1136/amiajnl-2010-000033. Epub 2011 Jul 1. PMID: 21724740; PMCID: PMC3197986. | Phil Gooch | 2011 |
| CPOE, CDSS | Hardenbol AX, Knols B, Louws M, Meulendijk M, Askari M. Usability aspects of medication-related decision support systems in the outpatient setting: A systematic literature review [published correction appears in Health Informatics J. 2018 Dec 17;:1460458218821466]. Health Informatics J. 2020;26(1):72-87. doi:10.1177/1460458218813732 | Alec Xander Hardenbol | 2018 |
| CDSS | Mollon B, Chong J Jr, Holbrook AM, Sung M, Thabane L, Foster G. Features predicting the success of computerized decision support for prescribing: a systematic review of randomized controlled trials. BMC Med Inform Decis Mak. 2009 Feb 11;9:11. doi: 10.1186/1472-6947-9-11. PMID: 19210782; PMCID: PMC2667396. | Brent Mollon | 2009 |
| Ariadne principles | Dinh TS, Brueckle MS, González-González AI, et al. Evidence-Based Decision Support for a Structured Care Program on Polypharmacy in Multimorbidity: A Guideline Upgrade Based on a Realist Synthesis. J Pers Med. 2022;12(1):69. Published 2022 Jan 7. doi:10.3390/jpm12010069 | Truc Sophia Dinh | 2022 |

**Table S3** – Use of each appropriateness tool according to the participants’ profession.

|  | **Geriatrician** | **General Pratictioner** | **Other MD** | **Pharmacist** | **Nurse** | **p-value** | **q-value*** |
| --- | --- | --- | --- | --- | --- | --- | --- |
| **Beers Criteria** |  |  |  |  |  | <0.001 | 0.003 |
| Used for each patient | 12 (21.05%) | 17 (11.97%) | 2 (3.57%) | 10 (4.37%) | 17 (9.83%) |  |  |
| Used for more than half of the patients | 18 (31.58%) | 24 (16.90%) | 7 (12.50%) | 17 (7.42%) | 8 (4.62%) |  |  |
| Used for less than half of the patients | 17 (29.82%) | 30 (21.13%) | 10 (17.86%) | 47 (20.52%) | 15 (8.67%) |  |  |
| Never used | 9 (15.79%) | 48 (33.80%) | 30 (53.57%) | 99 (43.23%) | 83 (47.98%) |  |  |
| Not known | 1 (1.75%) | 23 (16.20%) | 7 (12.50%) | 56 (24.45%) | 50 (28.90%) |  |  |
| **STOPP/START Criteria** |  |  |  |  |  | <0.001 | 0.003 |
| Used for each patient | 14 (24.56%) | 12 (8.45%) | 1 (1.79%) | 4 (1.75%) | 12 (6.94%) |  |  |
| Used for more than half of the patients | 18 (31.58%) | 21 (14.79%) | 9 (16.07%) | 14 (6.11%) | 12 (6.94%) |  |  |
| Used for less than half of the patients | 13 (22.81%) | 35 (24.65%) | 4 (7.14%) | 38 (16.59%) | 16 (9.25%) |  |  |
| Never used | 10 (17.54%) | 50 (35.21%) | 34 (60.71%) | 113 (49.34%) | 83 (47.98%) |  |  |
| Not known | 2 (3.51%) | 24 (16.90%) | 8 (14.29%) | 60 (26.20%) | 50 (28.90%) |  |  |
| **MULTIPAP/Ariadne principles** |  |  |  |  |  | <0.001 | 0.003 |
| Used for each patient | 5 (8.77%) | 5 (3.52%) | 1 (1.79%) | 1 (0.44%) | 14 (8.09%) |  |  |
| Used for more than half of the patients | 4 (7.02%) | 12 (8.45%) | 5 (8.93%) | 5 (2.18%) | 6 (3.47%) |  |  |
| Used for less than half of the patients | 9 (15.79%) | 25 (17.61%) | 5 (8.93%) | 19 (8.30%) | 7 (4.05%) |  |  |
| Never used | 23 (40.35%) | 69 (48.59%) | 32 (57.14%) | 122 (53.28%) | 87 (50.29%) |  |  |
| Not known | 16 (28.07%) | 31 (21.83%) | 13 (23.21%) | 82 (35.81%) | 59 (34.10%) |  |  |
| **CDSS** |  |  |  |  |  | <0.001 | 0.003 |
| Used for each patient | 10 (17.54%) | 44 (30.99%) | 5 (8.93%) | 9 (3.93%) | 30 (17.34%) |  |  |
| Used for more than half of the patients | 11 (19.30%) | 16 (11.27%) | 8 (14.29%) | 9 (3.93%) | 6 (3.47%) |  |  |
| Used for less than half of the patients | 10 (17.54%) | 25 (17.61%) | 6 (10.71%) | 26 (11.35%) | 11 (6.36%) |  |  |
| Never used | 20 (35.09%) | 43 (30.28%) | 31 (55.36%) | 119 (51.97%) | 76 (43.93%) |  |  |
| Not known | 6 (10.53%) | 14 (9.86%) | 6 (10.71%) | 66 (28.82%) | 50 (28.90%) |  |  |
| **Micromedex** |  |  |  |  |  | 0.010 | 0.059 |
| Used for each patient | 1 (1.75%) | 0 (0.00%) | 0 (0.00%) | 6 (2.62%) | 8 (4.62%) |  |  |
| Used for more than half of the patients | 4 (7.02%) | 3 (2.11%) | 2 (3.57%) | 5 (2.18%) | 7 (4.05%) |  |  |
| Used for less than half of the patients | 6 (10.53%) | 8 (5.63%) | 7 (12.50%) | 20 (8.73%) | 6 (3.47%) |  |  |
| Never used | 30 (52.63%) | 86 (60.56%) | 35 (62.50%) | 119 (51.97%) | 80 (46.24%) |  |  |
| Not known | 16 (28.07%) | 45 (31.69%) | 12 (21.43%) | 79 (34.50%) | 72 (41.62%) |  |  |
| **UpToDate** |  |  |  |  |  | <0.001 | 0.003 |
| Used for each patient | 6 (10.53%) | 5 (3.52%) | 4 (7.14%) | 7 (3.06%) | 11 (6.36%) |  |  |
| Used for more than half of the patients | 13 (22.81%) | 12 (8.45%) | 9 (16.07%) | 6 (2.62%) | 6 (3.47%) |  |  |
| Used for less than half of the patients | 22 (38.60%) | 27 (19.01%) | 17 (30.36%) | 33 (14.41%) | 11 (6.36%) |  |  |
| Never used | 11 (19.30%) | 67 (47.18%) | 23 (41.07%) | 102 (44.54%) | 76 (43.93%) |  |  |
| Not known | 5 (8.77%) | 31 (21.83%) | 3 (5.36%) | 81 (35.37%) | 69 (39.88%) |  |  |
| **INTERCheck web** |  |  |  |  |  | <0.001 | 0.003 |
| Used for each patient | 4 (7.02%) | 6 (4.23%) | 1 (1.79%) | 1 (0.44%) | 5 (2.89%) |  |  |
| Used for more than half of the patients | 10 (17.54%) | 10 (7.04%) | 2 (3.57%) | 9 (3.93%) | 4 (2.31%) |  |  |
| Used for less than half of the patients | 19 (33.33%) | 28 (19.72%) | 8 (14.29%) | 22 (9.61%) | 8 (4.62%) |  |  |
| Never used | 19 (33.33%) | 69 (48.59%) | 32 (57.14%) | 115 (50.22%) | 87 (50.29%) |  |  |
| Not known | 5 (8.77%) | 29 (20.42%) | 13 (23.21%) | 82 (35.81%) | 69 (39.88%) |  |  |

*Q-value stands for the p-value corrected for multiple testing through the Bonferroni correction. MD: medical doctor.

**Table S4** – Years of professional experiences (expressed as median (1^st^ quartile – 3^rd^ quartile)) and knowledge and usage patterns of appropriateness tool.

|  | **Used for each patient** | **Used for more than half of the patients** | **Used for less than half of the patients** | **Never used** | **Not known** | **p-value** | **q-value*** |
| --- | --- | --- | --- | --- | --- | --- | --- |
| **Beers Criteria** | 15 (7, 25) | 10 (4, 20) | 15 (6, 27) | 18 (8, 29) | 18 (7, 29) | 0.019 | 0.134 |
| **STOPP/START Criteria** | 15 (7, 23) | 10 (4, 22) | 12 (4, 29) | 18 (8, 27) | 17 (7, 30) | 0.006 | 0.043 |
| **MULTIPAP/Ariadne principles** | 18 (8, 30) | 12 (4, 21) | 12 (3, 30) | 18 (8, 28) | 15 (6, 27) | 0.10 | 0.687 |
| **CDSS** | 11 (3, 22) | 9 (2, 22) | 13 (3, 25) | 18 (9, 29) | 17 (8, 30) | <0.001 | <0.001 |
| **Micromedex** | 14 (7, 19) | 12 (2, 23) | 14 (7, 30) | 17 (6, 27) | 17 (6, 28) | 0.7 | 1 |
| **UpToDate** | 11 (6, 18) | 11 (3, 26) | 12 (5, 23) | 18 (7, 28) | 20 (8, 30) | <0.001 | 0.006 |
| **INTERCheck web** | 15 (5, 23) | 10 (4, 19) | 8 (3, 18) | 18 (9, 29) | 17 (7, 30) | <0.001 | <0.001 |

*Q-value stands for the p-value corrected for multiple testing through the Bonferroni correction. One invalid answer on the years of experience was removed from this analysis.

**Table S5 –** Other appropriateness tools used by participants

| **Tool** | **N (%)** |
| --- | --- |
| Websites/apps | 37 (11.97%) |
| Workplace information systems | 72 (23.30%) |
| National databases | 18 (5.83%) |
| AIFA website | 22 (7.12%) |
| Medical/scientific education | 13 (4.21%) |
| Guidelines | 10 (3.24%) |
| Interdisciplinary collaboration | 7 (2.27%) |
| Other | 5 (1.62%) |
| None | 125 (40.45%) |

Questionnaire – Italian version

Indagine sugli strumenti per migliorare appropriatezza prescrittiva e aderenza alla terapia

Gentile Collega,

L'Università di Bologna sta conducendo un'indagine in Emilia Romagna per comprendere quanto siano diffusi e utili gli strumenti a disposizione del personale sanitario per favorire l'appropriatezza della prescrizione medica e l'aderenza al trattamento farmacologico (esempio: criteri di inappropriatezza prescrittiva neII'anziano e relative applicazioni elettroniche, diario clinico per il paziente e sistemi di monitoraggio dell'assunzione dei medicinali).

Le domande che le verranno poste sono state predisposte sulla base di una mappatura della letteratura sui principali strumenti utilizzati dal personale sanitario a livello internazionale e validate da un piccolo campione di professionisti sanitari locali.

La sua partecipazione al questionario consentirà di comprendere come migliorare Io sviluppo di tali strumenti, la Ioro diffusione e la formazione degli operatori sanitari per un Ioro utilizzo più efficiente.

Il questionario è strutturato in affermazioni sulle quali fornire un'opinione in scala strutturata (esempio, da "moltissimo" a "per niente") e domande aperte. La preghiamo di completare il questionario in tutte le sue parti e nel modo più esaustivo possibile. La partecipazione richiederà circa 10 minuti del suo tempo e le risposte fornite saranno utilizzate solo per scopi di ricerca. I risultati saranno pubblicati in forma di articolo scientifico e saranno diffusi dalle istituzioni sanitarie locali deII'EmiIia Romagna. Sarà anche possibile ottenerli direttamente dal gruppo di ricerca dietro apposita richiesta all’indirizzo di posta elettronica riportato in fondo al messaggio. Questo studio rispetterà rigorosamente l’anonimato dei partecipanti.

Nel ringraziarla per la partecipazione, Le porgiamo Cordiali Saluti,

Elisabetta Poluzzi, Valentina Giunchi, Carlotta Lunghi, Giulia Grillini, Valentina Colonnello Dipartimento di Scienze Mediche e Chirurgiche

Alma Mater Studiorum - Università di Bologna Contatti: [elisabetta.poluzzi@unibo.it](mailto:elisabetta.poluzzi@unibo.it)

* Obbligatoria

1. Dopo aver preso visione dello scopo della ricerca e della modalità di utilizzo dei dati, le chiediamo il consenso alla partecipazione alla ricerca e all'utilizzo dei suoi dati in forma anonima *

- Ho compreso l’informativa e acconsento alla partecipazione
- Non partecipo (uscita dal modulo)

1. Genere *

- Maschio
- Femmina
- Altro/preferisco non specificarlo

1. Età *

- Meno di 40 anni
- Tra i 40 e i 60 anni
- Più di 60 anni

1. Professione *

- Medico di base
- Geriatra
- Farmacista
- Infermiere
- Altro

1. Indichi di seguito da quanti anni svolge la sua attuale professione. Se svolge la professione da meno di 1 anno, indichi "< 1" *
2. Considerando gli ultimi 12 mesi, in media, quanti pazienti vede ogni SETTIMANA? *

1. Considerando gli ultimi 12 mesi, in media, quanti pazienti di età superiore ai 65 anni, vede ogni SETTIMANA? *

# ADERENZA AL TRATTAMENTO

1. Per migliorare l'aderenza al trattamento farmacologico, a quanti pazienti over 65 ha consigliato negli ultimi 12 mesi l'utilizzo di: *

DIARIO CARTACEO DI AUTO-MONITORAGGIO DELLA TERAPIA?

- A nessun paziente
- A meno della metà dei pazienti
- A più della metà dei pazienti
- A tutti i pazienti
- Non Io conosco

1. Considerando la sua esperienza, quanto ritiene utile l'utilizzo del DIARIO CARTACEO DI AUTO-MONITORAGGIO DELLA TERAPIA per l'aderenza al trattamento? *

- Per niente
- Poco
- Molto
- Moltissimo

1. Quali sono, in base alla sua esperienza, le difficoltà e gli ostacoli legati all'utilizzo del DIARIO CARTACEO DI AUTO-MONITORAGGIO DELLA TERAPIA?
2. Pensa che nei prossimi 12 mesi inizierà o continuerà a suggerire l'utilizzo del DIARIO CARTACEO DI AUTO-MONITORAGGIO DELLA TERAPIA per migliorare l'aderenza del paziente? *

- Sicuramente no
- Probabilmente no
- Probabilmente sì
- Sicuramente sì

1. Per migliorare l'aderenza al trattamento farmacologico, a quanti pazienti over 65 ha consigliato negli ultimi 12 mesi l'utilizzo di: *

PORTAPILLOLE TRADIZIONALE CON SUDDIVISIONE GIORNALIERA?

- A nessun paziente
- A meno della metà dei pazienti
- A più della metà dei pazienti
- A tutti i pazienti
- Non Io conosco

1. Considerando la sua esperienza, quanto ritiene utile l'utilizzo del PORTAPILLOLE TRADIZIONALE CON SUDDIVISIONE GIORNALIERA per l'aderenza al trattamento? *

- Per niente
- Poco
- Molto
- Moltissimo

1. Quali sono, in base alla sua esperienza, le difficoltà e gli ostacoli legati all'utilizzo del PORTAPILLOLE TRADIZIONALE CON SUDDIVISIONE GIORNALIERA?
2. Pensa che nei prossimi 12 mesi inizierà o continuerà a suggerire l'utilizzo del PORTAPILLOLE TRADIZIONALE CON SUDDIVISIONE GIORNALIERA per migliorare l'aderenza del paziente? *

- Sicuramente no
- Probabilmente no
- Probabilmente sì
- Sicuramente sì

1. Per migliorare l'aderenza al trattamento farmacologico, a quanti pazienti over 65 ha consigliato negli ultimi 12 mesi l'utilizzo di: *

PORTAPILLOLE ELETTRONICO (CON PROMEMORIA E/O REGISTRAZIONE DELL'APERTURA)?

- A nessun paziente
- A meno della metà dei pazienti
- A più della metà dei pazienti
- A tutti i pazienti
- Non Io conosco

1. Considerando la sua esperienza, quanto ritiene utile l'utilizzo del PORTAPILLOLE ELETTRONICO (CON PROMEMORIA E/O REGISTRAZIONE DELL'APERTURA) per l'aderenza al trattamento? *

- Per niente
- Poco
- Molto
- Moltissimo

1. Quali sono, in base alla sua esperienza, le difficoltà e gli ostacoli legati all'utilizzo del PORTAPILLOLE ELETTRONICO (CON PROMEMORIA E/O REGISTRAZIONE DELL'APERTURA)?
2. Pensa che nei prossimi 12 mesi inizierà o continuerà a suggerire l'utilizzo del PORTAPILLOLE ELETTRONICO (CON PROMEMORIA E/O REGISTRAZIONE DELL'APERTURA) per migliorare l'aderenza del paziente?

- Sicuramente no
- Probabilmente no
- Probabilmente sì
- Sicuramente sì

1. Per migliorare l'aderenza al trattamento farmacologico, a quanti pazienti over 65 ha consigliato negli ultimi 12 mesi l'utilizzo di: *

SERVIZI DI MESSAGGISTICA (SMS o MMS) MHEALTH?

- A nessun paziente
- A meno della metà dei pazienti
- A più della metà dei pazienti
- A tutti i pazienti
- Non Io conosco

1. Considerando la sua esperienza, quanto ritiene utile l'utilizzo dei SERVIZI DI MESSAGGISTICA (SMS o MMS) MHEALTH per l'aderenza al trattamento? *

- Per niente
- Poco
- Molto
- Moltissimo

1. Quali sono, in base alla sua esperienza, le difficoltà e gli ostacoli legati all'utilizzo dei SERVIZI DI MESSAGGISTICA (SMS o MMS) MHEALTH?
2. Pensa che nei prossimi 12 mesi inizierà o continuerà a suggerire l'utilizzo di SERVIZI DI MESSAGGISTICA (SMS o MMS) MHEALTH per migliorare l'aderenza del paziente? *

- Sicuramente no
- Probabilmente no
- Probabilmente sì
- Sicuramente sì

1. Per migliorare l'aderenza al trattamento farmacologico, a quanti pazienti over 65 ha consigliato negli ultimi 12 mesi l'utilizzo di: *

SISTEMI DI RISPOSTA VOCALE INTERATTIVA (IVR)?

- A nessun paziente
- A meno della metà dei pazienti
- A più della metà dei pazienti
- A tutti i pazienti
- Non Io conosco

1. Considerando la sua esperienza, quanto ritiene utile l'utilizzo dei SISTEMI DI RISPOSTA VOCALE INTERATTIVA (IVR) per l'aderenza al trattamento? *

- Per niente
- Poco
- Molto
- Moltissimo

1. Quali sono, in base alla sua esperienza, le difficoltà e gli ostacoli legati all'utilizzo dei SISTEMI DI RISPOSTA VOCALE INTERATTIVA (IVR)?
2. Pensa che nei prossimi 12 mesi inizierà o continuerà a suggerire l'utilizzo di SISTEMI DI RISPOSTA VOCALE INTERATTIVA (IVR) per migliorare l'aderenza del paziente? *

- Sicuramente no
- Probabilmente no
- Probabilmente sì
- Sicuramente sì

1. Per migliorare l'aderenza al trattamento farmacologico, a quanti pazienti over 65 ha consigliato negli ultimi 12 mesi l'utilizzo di: *

SITI WEB EDUCATIVI E DI MONITORAGGIO TRAMITE FEEDBACK DA PARTE DI OPERATORI SANITARI?

- A nessun paziente
- A meno della metà dei pazienti
- A più della metà dei pazienti
- A tutti i pazienti
- Non Io conosco

1. Considerando la sua esperienza, quanto ritiene utile l'utilizzo dei SITI WEB EDUCATIVI E DI MONITORAGGIO TRAMITE FEEDBACK DA PARTE DI OPERATORI SANITARI per l'aderenza al trattamento? *

- Per niente
- Poco
- Molto
- Moltissimo

1. Quali sono, in base alla sua esperienza, le difficoltà e gli ostacoli legati all'utilizzo dei SITI WEB EDUCATIVI E DI MONITORAGGIO TRAMITE FEEDBACK DA PARTE DI OPERATORI SANITARI?
2. Pensa che nei prossimi 12 mesi inizierà o continuerà a suggerire l'utilizzo di SITI WEB EDUCATIVI E DI MONITORAGGIO TRAMITE FEEDBACK DA PARTE DI OPERATORI SANITARI per migliorare l'aderenza del paziente? *

- Sicuramente no
- Probabilmente no
- Probabilmente sì
- Sicuramente sì

1. Per migliorare l'aderenza al trattamento farmacologico, a quanti pazienti over 65 ha consigliato negli ultimi 12 mesi l'utilizzo di: *

APP EDUCATIVE CON FUNZIONE DI PROMEMORIA E/O CHE CONSENTONO DI COMUNICARE CON IL MEDICO CURANTE 0 ALTRO PERSONALE SANITARIO?

- A nessun paziente
- A meno della metà dei pazienti
- A più della metà dei pazienti
- A tutti i pazienti
- Non Io conosco

1. Considerando la sua esperienza, quanto ritiene utile l'utilizzo di APP EDUCATIVE CON FUNZIONE DI PROMEMORIA E/O CHE CONSENTONO DI COMUNICARE CON IL MEDICO CURANTE 0 ALTRO PERSONALE SANITARIO per l'aderenza al trattamento? *

- Per niente
- Poco
- Molto
- Moltissimo

1. Quali sono, in base alla sua esperienza, le difficoltà e gli ostacoli legati all'utilizzo di APP EDUCATIVE CON FUNZIONE DI PROMEMORIA E/O CHE CONSENTONO DI COMUNICARE CON IL MEDICO CURANTE 0 ALTRO PERSONALE SANITARIO?
2. Pensa che nei prossimi 12 mesi inizierà o continuerà a suggerire l'utilizzo di APP EDUCATIVE CON FUNZIONE DI PROMEMORIA E/O CHE CONSENTONO DI COMUNICARE CON IL MEDICO CURANTE 0 ALTRO PERSONALE SANITARIO per migliorare l'aderenza del paziente? *

- Sicuramente no
- Probabilmente no
- Probabilmente sì
- Sicuramente sì

# APPROPRIATEZZA PRESCRITTIVA

1. Per favorire l'APPROPRIATEZZA dei trattamenti farmacologici neII'anziano è possibile far riferimento ai criteri BEERS, una lista di farmaci suddivisa in tre sezioni:

- farmaci da evitare
- farmaci potenzialmente inappropriati in determinate condizioni
- farmaci da utilizzare con cautela

Per maggiori informazioni: By the 2019 American Geriatrics Society Beersb criteria Update Expert Panel. American Geriatrics Society 2019 Updated AGS Beers Criteria for Potentially Inappropriate Medication Use in Older

Adults. Journal o[the American Geriatrics Society vol. 67,4 (2019): 674-694. doi:10.1 1 1 1/jgs.15767

Negli ultimi 1 2 mesi, si è avvalso/a dei criteri di BEERS

- Per nessun paziente
- Per meno della metà dei pazienti
- Per più della metà dei pazienti
- Per tutti i pazienti
- Non li conosco

1. Per favorire l’APPROPRIATEZZA nella prescrizione è possibile seguire i criteri START/STOPP.

I criteri STOPP (Screening Tool of Older Person’s Prescriptions) identificano farmaci da evitare neII‘anziano perché non adatti per posologia o durata della terapia.

I criteri START (Screening Tool to Alert Doctor to Right Treatment) identificano farmaci con chiaro beneficio in presenza di una precisa diagnosi neII'anziano.

Per maggiori informazioni: Diaz Planelles I, et al. Prevalence of Potentially Inappropriate Prescriptions According to the New STOPP/START Criteria in Nursing Homes: A Systematic Review. Healthcare (Basel). 2023;1 1(3):422. Published 2023 Feb 1. doi:10.3390/heaIthcare1 1030422

Negli ultimi 1 2 mesi, si è avvalso/a dei criteri di START/STOPP

- Per nessun paziente
- Per meno della metà dei pazienti
- Per più della metà dei pazienti
- Per tutti i pazienti
- Non li conosco

1. Per favorire l’APPROPRIATEZZA PRESCRITTIVA si possono seguire linee guida e protocolli di pratica clinica come MULTIPAP (Improving Healthcare in Multimorbidity and Polypharmacy in Primary Care - Principi di Ariadne).

Per maggiori informazioni: Del Cura-Gonzàlez I, et al. How to Improve Healthcare for Patients with Multimorbidity and Polypharmacy in Primary Care: A Pragmatic Cluster-Randomized Clinical Trial of the MULTIPAP Intervention. 7 Pers Med. 2022;12(5):752. Published 2022 May 6. doi:10.3390/jpm12050752

Negli ultimi 1 2 mesi ha seguito questi protocolli *

- Per nessun paziente
- Per meno della metà dei pazienti
- Per più della metà dei pazienti
- Per tutti i pazienti
- Non li conosco

1. Per favorire l’APPROPRIATEZZA PRESCRITTIVA sono utili software di supporto alla prescrizione, ossia programmi informatici che consentono la ricettazione elettronica a partire dalla cartella clinica elettronica del paziente e che possono includere raccomandazioni sulle terapie farmacologiche per Io specifico paziente (tali raccomandazioni si basano su algoritmi che derivano da linee guida e criteri di appropriatezza/inappropriatezza, si veda anche domande 36-38).

Negli ultimi 1 2 mesi si è avvalso/a di tali strumenti *

- Per nessun paziente
- Per meno della metà dei pazienti
- Per più della metà dei pazienti
- Per tutti i pazienti
- Non li conosco

1. Per favorire l’APPROPRIATEZZA PRESCRITTIVA possono essere utilizzati numerosi database contenenti informazioni evidence-based su farmaci, Ioro profilo di sicurezza, rischio di interazioni, eventuali monitoraggi utili.

Negli ultimi 1 2 mesi si è avvalso/a di *

|  | | Per meno | Per più |  | |
| --- | --- | --- | --- | --- | --- |
|  |  | della metà | della metà |  |  |
|  | Per nessun | dei | dei | Per tutti i | Non Io |
| Micromedex | paziente  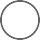 | pazienti  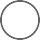 | pazienti  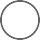 | pazienti  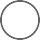 | conosco  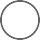 |
| UpToDate | 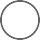 | 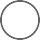 | 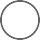 | 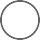 | 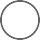 |

1. Per favorire l’APPROPRIATEZZA PRESCRITTIVA può essere utilizzato INTERCheck, un sistema di supporto alle prescrizioni che ha l'obiettivo di bilanciare rischi e benefici di una terapia fornendo un un punteggio di rischio di potenziali interazioni farmacologiche.

Per maggiori informazioni: <https://intercheckweb.marionegri.it/>

Negli ultimi 12 mesi si è avvalso/a di tale strumento

- Per nessun paziente
- Per meno della metà dei pazienti
- Per più della metà dei pazienti
- Per tutti i pazienti
- Non Io conosco

1. Negli ultimi 1 2 mesi, di quali ulteriori strumenti si è avvalso/a?
2. Ci sono stati specifici ostacoli all'utilizzo degli strumenti per favorire l'appropriatezza che ha utilizzato? (le chiediamo di definire gli ostacoli specifici per singolo strumento utilizzato)
3. Cosa potrebbe essere utile per supportarla nell'attività di monitoraggio deII'APPROPRIATEZZA PRESCRITTIVA in futuro?
4. Frequenterebbe un corso o seguirebbe un tutorial di circa 1 ora sugli strumenti a disposizione? *

Assolutamente Probabilmente Probabilmente Assolutamente no no sì si


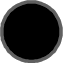

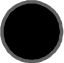

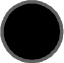

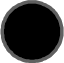
Per l’aderenza

al trattamento


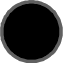

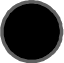

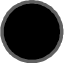

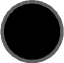
Per l’appropriatez za

prescrittiva

1. La ringraziamo per la partecipazione. Se Io desidera, può utilizzare questo spazio per condividere un'opinione o un commento sul questionario o su aspetti che non sono stati affrontati nella presente indagine e che ritiene utili per l'integrazione alle risposte già fornite.

Gentile collega, la sua esperienza sarebbe un contributo prezioso per la nostra ricerca

Siamo consapevoli che la sua professione richiede un impegno costante.

La ringraziamo in ogni caso per aver preso in considerazione il nostro questionario e rispettiamo la sua scelta di non partecipare.

Grazie per aver dedicato il suo tempo a questa ricerca.

Desideriamo esprimerle la nostra più sincera gratitudine per aver collaborato allo studio.

Questo contenuto non è stato creato né approvato da Microsoft. I dati che invii verranno recapitati al proprietario del modulo.


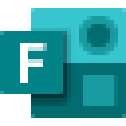
Microsoft Forms

**Survey on Tools to Improve Prescriptive Appropriateness and Adherence to Therapy**

Dear Colleague,

The University of Bologna is conducting a survey in the Emilia-Romagna region to assess the prevalence and effectiveness of tools available to healthcare professionals for promoting appropriate medical prescriptions and adherence to pharmacological treatments (e.g., criteria for prescribing appropriateness in older adults and related electronic applications, clinical diaries for patients, and systems for monitoring medication intake).

The survey questions have been developed based on a literature review of the primary tools used by healthcare professionals internationally and have been validated by a small sample of local healthcare professionals.

Your participation in this questionnaire is invaluable in helping us understand how to improve the development and dissemination of these tools, as well as enhance the training of healthcare professionals to optimize their use.

The questionnaire consists of statements rated on a structured scale (e.g., from “very much” to “not at all”) and open-ended questions. Please complete the questionnaire in full and provide responses as comprehensively as possible. Completing the survey will take approximately 10 minutes of your time, and all responses will be used solely for research purposes.

The results of this study will be published as a scientific article and disseminated by local health institutions in Emilia-Romagna. Additionally, they can be obtained directly by contacting the research team at the email address provided below. This study fully guarantees the anonymity of all participants.

Thank you for your participation.

Best regards,

Thank you for your participation.

Best regards,

Elisabetta Poluzzi, Valentina Giunchi, Carlotta Lunghi, Giulia Grillini, Valentina Colonnello Department of Medical and Surgical Sciences

Alma Mater Studiorum – University of Bologna

Contat: elisabetta.poluzzi@unibo.it

* Mandatory

1. After reviewing the purpose of the research and the data usage method, we ask for your consent to participate in the research and to use your data anonymously *

- I have read the information and consent to participate
- I do not consent to participate (exit the form)

1. Gender *

- Male
- Female
- Other/Prefer not to specify

1. Age *

- Under 40 years
- Between 40 and 60 years
- Over 60 years

1. Profession *

- General practitioner
- Geriatrician
- Pharmacist
- Nurse
- Other

1. Indicate below how many years you have been in your current profession. If you have been in the profession for less than 1 year, please indicate "< 1" *
2. Considering the last 12 months, on average, how many patients do you see per week? *
3. Considering the last 12 months, on average, how many patients over 65 years of age do you see per week? *

# **ADHERENCE TO TREATMENT**

1. To improve adherence to pharmacological treatment, haw many patients over 65 have you recommended in the last months to use the following tools?

PAPER DIARY FOR SELF-MONITORING OF THERAPY *

- None
- Fewer than half of the patients
- More than half of the patient
- All patients
- I am not familiar with it

1. Based on your experience, how useful do you consider the use of the

PAPER DIARY FOR SELF-MONITORING OF THERAPY in improving treatment

adherence? *

- Not at all
- Slightly
- Very
- Extremely

1. Based on your experience, what are the difficulties and obstacles related to the use of the PAPER DIARY FOR SELF-MONITORING OF THERAPY?

11. Do you think you will start or continue recommending the use of PAPER DIARY FOR SELF-MONITORING OF THERAPY in the next 12 months to improve treatment adherence? *

- Definitely not
- Probably no t
- Probably yes
- Definitely yes

1 2. To improve adherence to pharmacological treatment, haw many patients over 65 have you recommended in the last months to use the following instrument:

TRADITIONAL PILLBOX WITH DAILY DIVISION? *

- None
- Fewer than half of the patients
- More than half of the patient
- All patients
- I am not familiar with it

1. Based on your experience, how useful do you consider the use of the TRADITIONAL PILLBOX WITH DAILY DIVISION to improve treatment adherence? *

- Not at all
- Slightly
- Very
- Extremely

1. Based on your experience, what are the difficulties and obstacles related to the use of the TRADITIONAL PILLBOX WITH DAILY DIVISION?
2. Do you think you will start or continue recommending the use of TRADITIONAL PILLBOX WITH DAILY DIVISION in the next 12 months to improve patient adherence? *

- Definitely not
- Probably no t
- Probably yes
- Definitely yes

1. To improve adherence to pharmacological treatment, haw many patients over 65 have you recommended in the last months to use the following instrument:

ELECTRONIC PILLBOX (WITH REMINDER AND/OR RECORDING OF OPENING)? *

- None
- Fewer than half of the patients
- More than half of the patient
- All patients
- I am not familiar with it

1. Based on your experience, how useful do you consider the use of the ELECTRONIC PILLBOX (WITH REMINDER AND/OR RECORDING OF OPENING) to improve treatment adherence? *

- Not at all
- Slightly
- Very
- Extremely

1. Based on your experience, what are the difficulties and obstacles related to the use of the ELECTRONIC PILLBOX (WITH REMINDER AND/OR RECORDING OF OPENING)?
2. Do you think you will start or continue recommending the use of ELECTRONIC PILLBOX (WITH REMINDER AND/OR RECORDING OF OPENING) in the next 12 months to improve patient adherence? *

- Definitely not
- Probably no t
- Probably yes
- Definitely yes

1. To improve adherence to pharmacological treatment, haw many patients over 65 have you recommended in the last months to use the following instrument:

MHEALTH MESSAGE SERVICES (SMS or MMS)? *

- None
- Fewer than half of the patients
- More than half of the patient
- All patients
- I am not familiar with it

1. Based on your experience, how useful do you consider the use of the MHEALTH MESSAGE SERVICES (SMS or MMS) to improve treatment adherence? *

- Not at all
- Slightly
- Very
- Extremely

1. Based on your experience, what are the difficulties and obstacles related to the use of the MHEALTH MESSAGE SERVICES (SMS or MMS)?
2. Do you think you will start or continue recommending the use of MHEALTH MESSAGE SERVICES (SMS or MMS) in the next 12 months to improve patient adherence? *

- Definitely not
- Probably no t
- Probably yes
- Definitely yes

1. To improve adherence to pharmacological treatment, haw many patients over 65 have you recommended in the last months to use the following instrument:

INTERACTIVE VOICE RESPONSE SYSTEMS (IVR)? *

- None
- Fewer than half of the patients
- More than half of the patient
- All patients
- I am not familiar with it

1. Based on your experience, how useful do you consider the use of the INTERACTIVE VOICE RESPONSE SYSTEMS (IVR) to improve treatment adherence? *

- Not at all
- Slightly
- Very
- Extremely

1. Based on your experience, what are the difficulties and obstacles related to the use of the INTERACTIVE VOICE RESPONSE SYSTEMS (IVR)?
2. Do you think you will start or continue recommending the use of INTERACTIVE VOICE RESPONSE SYSTEMS (IVR) in the next 12 months to improve patient adherence? *

- Definitely not
- Probably no t
- Probably yes
- Definitely yes

1. To improve adherence to pharmacological treatment, how many patients over 65 have you recommended in the last months to use the following instrument:

EDUCATIONAL AND MONITORING WEBSITES THROUGH FEEDBACK FROM HEALTH PROFESSIONALS? *

- None
- Fewer than half of the patients
- More than half of the patient
- All patients
- I am not familiar with it

1. Based on your experience, how useful do you consider the use of the EDUCATIONAL AND MONITORING WEBSITES THROUGH FEEDBACK FROM HEALTH PROFESSIONALS to improve treatment adherence? *

- Not at all
- Slightly
- Very much
- Extremely

1. Based on your experience, what are the difficulties and obstacles related to the use of the EDUCATIONAL AND MONITORING WEBSITES THROUGH FEEDBACK FROM HEALTH PROFESSIONALS?
2. Do you think you will start or continue recommending the use of EDUCATIONAL AND MONITORING WEBSITES THROUGH FEEDBACK FROM HEALTH PROFESSIONALS in the next 12 months to improve patient adherence? *

- Definitely not
- Probably no t
- Probably yes
- Definitely yes

1. To improve adherence to pharmacological treatment, haw many patients over 65 have you recommended in the last months to use the following instrument:

EDUCATIONAL APPS WITH A REMINDER FUNCTION AND/OR COMMUNICATION WITH THE ATTENDING PHYSICIAN OR OTHER HEALTH PERSONNEL? *

- None
- Fewer than half of the patients
- More than half of the patient
- All patients
- I am not familiar with it

1. Based on your experience, how useful do you consider the use of the EDUCATIONAL APPS WITH A REMINDER FUNCTION AND/OR COMMUNICATION WITH THE ATTENDING PHYSICIAN OR OTHER HEALTH PERSONNEL to improve treatment adherence? *

- Not at all
- Slightly
- Very
- Extremely

1. Based on your experience, what are the difficulties and obstacles related to the use of the EDUCATIONAL APPS WITH A REMINDER FUNCTION AND/OR COMMUNICATION WITH THE ATTENDING PHYSICIAN OR OTHER HEALTH PERSONNEL?
2. Do you think you will start or continue recommending the use of EDUCATIONAL APPS WITH A REMINDER FUNCTION AND/OR COMMUNICATION WITH THE ATTENDING PHYSICIAN OR OTHER HEALTH PERSONNEL in the next 12 months to improve patient adherence? *

- Definitely not
- Probably no t
- Probably yes
- Definitely yes

**Prescriptive Appropriateness**

1. To promote the PRESCRIBING APPROPRIATENESS of pharmacological treatments in older adults, the BEERS CRITERIA can be referenced. This is a list of medications divided into three sections:
   -Medications to avoid
   -Medications potentially inappropriate in certain conditions
   -Medications to use with caution

For more information:

By the 2019 American Geriatrics Society Beers Criteria Update Expert Panel. American Geriatrics Society 2019 Updated AGS Beers Criteria for Potentially Inappropriate Medication Use in Older Adults.

*Journal o[the American Geriatrics Society* vol. 67,4 (2019): 674-694. doi:10.1 1 1 1/jgs.15767

In the last 12 months, have you used BEERS CRITERIA? *

- For no patients
- For fewer than half of the patients
- For more than half of the patients
- For all patient
- I am not familiar with them

1. To promote PRESCRIBING APPROPRIATENESS in prescribing, the START/STOPP CRITERIA can be followed.
   The STOPP CRITERIA (Screening Tool of Older Person’s Prescriptions) identify medications to avoid in older adults due to inappropriate dosage or duration of therapy.
   The START criteria (Screening Tool to Alert Doctor to Right Treatment) identify medications with clear benefits for older adults with specific diagnoses.

For more information:

Diaz Planelles I, et al. Prevalence of Potentially Inappropriate Prescriptions According to the New STOPP/START Criteria in Nursing Homes: A Systematic Review. *Healthcare (Basel).* 2023;1 1(3):422.

Published 2023 Feb 1. doi:10.3390/heaIthcare1 1030422

In the last 12 months, have you used STOPP/START CRITERIA? *

- For no patients
- For fewer than half of the patients
- For more than half of the patients
- For all patient
- I am not familiar with them

## To promote PRESCRIBING APPROPRIATENESS, clinical practice guidelines and protocols such as MULTIPAP (Improving Healthcare in Multimorbidity and Polypharmacy in Primary Care - Ariadne Principles) can be followed.

For more information:

Del Cura-Gonzàlez I, et al. How to Improve Healthcare for Patients with Multimorbidity and Polypharmacy in Primary Care: A Pragmatic Cluster-Randomized Clinical Trial of the MULTIPAP Intervention. 7 *Pers Med.*

2022;12(5):752. Published 2022 May 6. doi:10.3390/jpm12050752

In the last 12 months, have you used MULTIPAP? *

- For no patients
- For fewer than half of the patients
- For more than half of the patients
- For all patient
- I am not familiar with it

1. To promote PRESCRIBING APPROPRIATENESS, prescribing SUPPORT SOFTWARE can be useful. These are computer programs that allow electronic prescriptions based on the patient's electronic health record and may include recommendations for pharmacological therapies tailored to the specific patient (such recommendations are based on algorithms derived from guidelines and appropriateness/inappropriateness criteria, see also questions 36-38).

In the last 12 months, have you used SUPPORT SOFTWARE? *

- For no patient
- For fewer than half of the patients
- For more than half of the patients
- For all patients
- I am not familiar with them

1. To promote APPROPRIATE PRESCRIBING, several DATABASES containing evidence-based information about medications, their safety profiles, potential interactions, and relevant monitoring requirements can be utilized.

In the last 12 months, which of the following DATABASES have you used? *

|  | |  |  |  | |
| --- | --- | --- | --- | --- | --- |
|  |  |  |  |  |  |
|  | For no patient | For less than half | For more than half | For all patient | Not known |
| Micromedex | 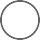 | 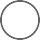 | 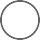 | 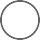 | 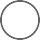 |
| UpToDate | 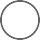 | 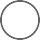 | 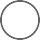 | 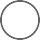 | 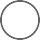 |

1. To promote PRESCRIBING APPROPRIATENESS, INTERCheck WEB can be useful.

INTERCheck is a prescribing support system that helps balance the risks and benefits of a therapy by providing a risk score for potential drug interactions.

For more information: https://intercheckweb.marionegri.it/

In the last 12 months, have you used SUPPORT SOFTWARE? *

- For no patient
- For fewer than half of the patients
- For more than half of the patients
- For all patients
- I am not familiar with them

1. What other prescribing tools have you used in the last 12 months?
2. Have you encountered specific challenges or obstacles when using the tools to promote appropriateness? (Please specify the challenges for each tool used)
3. What support would help you monitor APPROPRITE PRESCRIBING in the future?

Would you attend a course or follow a tutorial (approximately 1 hour) on the tools available? *

Definitely not Probably not Probably yes Definitely yes


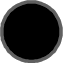

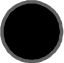

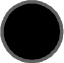

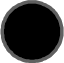
For Adherence


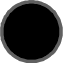

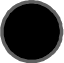

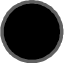

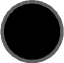
For Appropriateness

1. Thank you for your participation. If you wish, you can use this space to share your opinion or comment on the questionnaire or on aspects not addressed in this survey that you consider useful to complement the responses provided.

Dear colleague,

Your experience is a valuable contribution to our research. We are aware that your profession demands constant dedication.

We thank you for considering our questionnaire and respect your choice not to participate.

Thank you for taking the time to contribute to this research.

We wish to express our sincere gratitude for collaborating in the study.

This content was neither created nor approved by Microsoft. The data you submit will be delivered to the form owner.


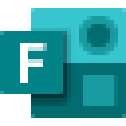
Microsoft Forms
